# Unlocking Novel Therapeutic Potential of Angiotensin II Receptor Blockers

**DOI:** 10.3390/ijms26188819

**Published:** 2025-09-10

**Authors:** Filippos Panteleimon Chatzipieris, Kiriaki Mavromoustakou, John M. Matsoukas, Thomas Mavromoustakos

**Affiliations:** 1 Laboratory of Organic Chemistry, Department of Chemistry, National and Kapodistrian University of Athens, 15771 Athens, Greece; fchatzip@chem.uoa.gr; 2 First Cardiology Department, Medical School, National and Kapodistrian University of Athens, Hippokration General Hospital, 11527 Athens, Greece; mavromoustakoukiriaki@yahoo.gr; 3 Institute for Health and Sport, Victoria University, Melbourne, VIC 3030, Australia; imats@upatras.gr; 4 Department of Physiology and Pharmacology, Cumming School of Medicine, University of Calgary, Calgary, AB T2N 4N1, Canada; 5 NewDrug, P.C., Patras Science Park, 26504 Patras, Greece; 6 Department of Chemistry, University of Patras, 26504 Patras, Greece

**Keywords:** hypertension (HT), drug repurposing (repositioning, reprofiling, re-tasking, rediscovery, rescue), renin–angiotensin–aldosterone system (RAAS), neurodegenerative diseases, AT1 antagonists, bisartans, sartan derivatives

## Abstract

Pharmaceutical companies keep producing novel drugs and drug treatments for improving the life of every sick individual, most often following a pattern; a specific drug for a specific condition. Evidence suggests that different medications can have a positive effect on different pathological conditions. The full potential of existing therapies can be revealed through drug repurposing—also referred to as drug repositioning, reprofiling, or re-tasking—which involves identifying new therapeutic uses for approved or investigational drugs beyond their original indications. One significant target in this context is the renin–angiotensin–aldosterone system (RAAS), a crucial regulator of blood pressure and fluid homeostasis, and a central focus in the treatment of chronic cardiovascular conditions such as arterial hypertension (AH) and heart failure (HF). Interestingly, novel investigations show that AT1 antagonists (sartans) are able to broaden their therapeutic scope and potentially combat other diseases such as neurodegenerative diseases, cancer, and osteoarthritis, and even help people with methamphetamine and opioid addiction.

## 1. Introduction

### 1.1. Introducing Drug Repurposing

Drug repurposing (repositioning, reprofiling, re-tasking, rediscovery, or rescue) is a method used to discover new therapeutic applications for approved or investigational drugs beyond their original medical purposes [1,2]. This approach provides several benefits compared to creating a completely new drug for the same condition. A repurposed drug has an advantage because it has been found safe in preclinical and early human trials [3,4]. As a result, they are less likely to fail due to safety concerns in later efficacy trials. Also, the drug development timeline can be shortened since much of the preclinical testing, safety evaluation, and sometimes formulation work has already been carried out [5,6]. Finally, the required investment is generally lower, although the amount can vary significantly based on the development stage and process of the repurposing candidate. Combined, these benefits can lead to a quicker and less risky return on investment when developing repurposed drugs, with overall lower costs after considering potential failures. Most importantly, repurposed drugs might uncover new targets and biological pathways that could be explored further [7,8].

### 1.2. RAAS Physiology

The renin–angiotensin–aldosterone system (RAAS) is a key regulator of vascular tone and fluid homeostasis. Juxtaglomerular cells release renin which initiates the conversion of angiotensinogen to angiotensin I (Ang I) which is then converted to angiotensin II (Ang II) by the angiotensin-converting enzyme (ACE) and other enzymes [9]. Ang II acts predominantly via angiotensin II type 1 (AT1) receptors to raise blood pressure through vasoconstriction, sympathetic activation, and enhanced sodium retention. Ang II also stimulates aldosterone secretion, further promoting sodium reabsorption [10]. Chronic AT1 activation contributes to vascular inflammation, hypertrophy, and fibrosis. In the opposite direction, AT2 receptor engagement counteracts these effects through vasodilation and natriuresis. In particular, in the RAS, angiotensin-converting enzyme 2 (ACE2) cleaves vasoconstrictor Ang II to delete Phe at position 8 and produce vasodilator heptapeptide Ang (1–7), counterbalancing the toxic axis ACE1/Ang II, maintaining homeostasis, and controlling blood regulation. An example of the beneficial effect of the protective Ang (1–7)/MAS (MAS proto-oncogene) axis in the renin–angiotensin system is its heptapeptide constituent alamandine which reverses vascular dysfunction [11]. Pharmacologic blockade of RAAS offers a therapeutic strategy across multiple cardiovascular and renal conditions [12].

### 1.3. Multi-Target Directed Ligand (MTDL) Approach

Targeting several pathways at once has proven effective in improving drug performance and combating resistance, particularly in complex diseases. From Alzheimer’s to cancer, more and more data show that most of the publicly known diseases are actually multifactorial. The strategy of “one molecule for one drug target”, while having shown improvements in the lives of many people worldwide, is outdated. Focus should also be given to the development of Multi-Target Directed Ligands (MTDLs) with the simplest example being dual inhibitors. MTDLs are rationally designed to act on several targets and have become a widely accepted approach in drug discovery [13,14,15,16].

**Figure 1:**
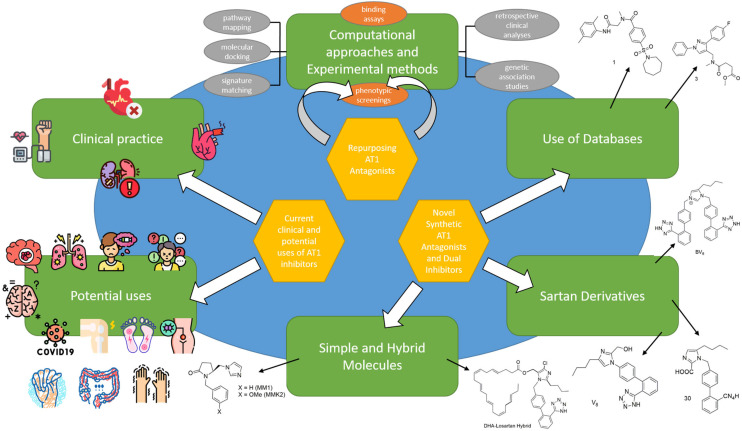
A graphical representation summarizing the topics that will be analyzed in this review article. Specifically, we will start by listing methodologies in which drug repurposing can be achieved (computationally and experimentally). Then, we will examine how drugs that act as AT1 receptor inhibitors can also exert their effects in other pathologies. Specifically, we will start with the ‘core’ diseases that often coexist in patients taking these drugs (HT; hypertension, HF; heart failure, CKD; chronic kidney disease, ACS; acute coronary syndrome). Then, we will list the diseases in which the RAAS plays an important role and therefore inhibition of AT1 receptors could help in the treatment of patients as indicated in research (AD; Alzheimer’s disease, PD; Parkinson’s disease, RA; rheumatoid arthritis, OA; osteoarthritis, DN; diabetic nephropathy, DPN; diabetic peripheral neuropathy, SARS-CoV-2, CS; candidosis, FS; fibrosis, TFSSc; tissue fibrosis in systemic sclerosis, GC; glioma cancer, MS; Marfan syndrome, IBDs; inflammatory bowel diseases, AT; anxiety, PI; pathogenic inflammation, OPA; opioid addiction). Finally, we highlight the work of our laboratory and collaborators towards the design, synthesis, and pharmacological evaluation of innovative bioactive compounds, some of which are also multifunctional drugs, for the treatment of hypertension and other diseases. Such compounds include simple molecules, hybrid molecules, sartan derivatives, and compounds derived from cheminformatic databases.

Thus, the focus of this review will be on the study of molecules acting as ARBs and their potential use in other pathological conditions as well as their ability to inhibit multiple molecular targets. Moreover, we will present our laboratory’s work on the development of novel AT1 inhibitors and dual inhibitors for the treatment of multiple diseases. The graphic representation in Figure 1 summarizes the topics covered in this review article.

## 2. Establishing Drug Repurposing: The Example of Sildenafil

Newly developed drugs do not enter the market until all regulatory conditions have been fulfilled [17]. For a drug candidate to be established as a treatment for a certain disease, much time (10–15 years) [18], money (roughly USD 1.8 billion until its entry to the market) [19], and effort is needed. The first step is to design and synthesize the compound of interest. Testing its in vitro and in vivo activities is the second step of drug development, along with the examination of its toxicity effects [20,21]. By the time it proceeds to clinical trials, a significant amount of money has been consumed for the purchase of resources, while many experimental animals have also been used, which is not ideal from a bioethical point of view. A further rise in costs is observed when scaling up the production [2]. Due to these limitations, the research community draws its attention to established drugs, which have already passed the standards set by the strict regulatory authorities and have been deemed safe for use. For a repurposed molecule, existing safety, preclinical, and efficacy data are already available, allowing researchers to make well-informed decisions at every stage of drug development [4,22]. Investigational molecules that do not demonstrate efficacy for their initial indication are also promising candidates. Such compounds can be redirected toward new indications and ultimately developed into effective therapies, a strategy especially valuable for rare diseases, which pose substantial challenges in terms of diagnosis, treatment, and limited resources [23,24]. This strategy helps mitigate the rising costs of drug development, thereby reducing patients’ out-of-pocket expenses and ultimately lowering the overall cost of therapy [4,22,24,25].

The best example of a drug being developed to regulate one condition and ending up more well-known and profitable to another is sildenafil (Viagra). In 1986, Pfizer established a project team of scientists in Sandwich, UK to develop a selective phosphodiesterase-5 (PDE5) inhibitor and assess its preclinical pharmacological properties. The goal was simple: the inhibition of PDE5, a selective catalyst in the breakdown of cyclic guanosine monophosphate (cGMP), shall result in the excess of this molecule in the body, which will subsequently promote the initiation of a cascade of reactions that ultimately decreases intracellular calcium levels, thereby promoting relaxation of the smooth muscle. Thus, it would be a mixed dilator of arteries and veins by relaxing vascular smooth muscle, which lowers peripheral vascular resistance and cardiac preload while improving blood flow to ischemic heart tissue. This would have an anti-anginal effect. In 1992, when given doses up to 75 mg three times daily for 10 days, some volunteers experienced side effects such as headaches, flushing, indigestion, and muscle pain. Additionally, some reported penile erections as an unexpected effect [26]. Based on observations and studies of Ignarro et al. [27] in the early 1990s, where it was found that nitric oxide (NO) acts as the neurotransmitter released from cavernous nerves during sexual arousal, triggering cGMP production and ultimately leading to an erection [27], clinical trials began in late 1993 for patients with erectile dysfunction [26] and the rest is history.

Success stories only begin with the example of sildenafil. Table 1 presents several successful cases, showing both their original indications and, in most instances, their newly approved indications.

Sildenafil is the best example of retrospective clinical analysis leading to repurposing (or rescue if the drug had otherwise failed for its primary indication) of a candidate molecule. Typically, a drug-repurposing strategy involves three steps before advancing a candidate drug through the development pipeline. The first step is identifying a potential molecule for a specific indication, also known as hypothesis generation. The second step involves assessing the drug’s mechanism of action using preclinical models. The third step is to evaluate the drug’s efficacy in phase II clinical trials, provided there is adequate safety data from phase I studies conducted for the original indication. Among these three steps, the first—accurately identifying the appropriate drug for a specific indication with high confidence—is crucial. This is the stage where modern methods for hypothesis generation can be particularly valuable. These systematic strategies are categorized into computational and experimental approaches, and their combined use is becoming increasingly common to enhance effectiveness. The most frequently employed computational methods include retrospective clinical analyses, molecular docking, signature matching, genetic association studies, pathway mapping, and the use of emerging data sources, while experimental approaches such as binding assays to identify relevant target interactions and phenotypic screenings are also employed [2,22,143].

Although drug repurposing as a strategy is advantageous due to its cost-effectiveness and ability to shorten the drug-discovery timeline, it is important to carefully consider the various factors that may influence its implementation. A major barrier is the pharmaceutical industry’s limited focus on financially rewarding diseases, which reduces opportunities for drug repurposing in orphan diseases and neglected tropical disorders. In addition, repurposed drugs face legal challenges such as limited patent protection and difficulties in conducting clinical trials, both of which reduce their chances of successful development. It is crucial to address further challenges, including the risk of false-positive signals during data mining and the susceptibility of hypothesis validation to bias and confounding factors. The lack of clear regulatory guidance represents an additional obstacle in advancing drug-repurposing efforts [144]. Moreover, the repurposed drugs might have implications with off-target interactions, resulting in adverse effects. For instance, sildenafil can cause headache, flushing, nasal congestion, dyspepsia, and a slight decrease in systolic and diastolic blood pressure. Moreover, combining the drug with alcohol can lead to cerebrovascular hemorrhage; a rare fatal side effect [145].

## 3. Repurposing Angiotensin II Receptor Blockers (ARBs)

ARBs are used as first-line treatment, with different class indications, in hypertension, heart failure, post-myocardial infarction, chronic kidney disease and for the prevention of cardiovascular events, particularly in individuals with atherosclerotic coronary artery disease (CAD) or type 2 diabetes with damage to at least one organ [146].

### 3.1. Hypertension

Pioneer research on angiotensin and the mechanism that triggers hypertension and blood pressure has revealed a Charge Relay System (CRS) mechanism, analogous to serine proteases, involving the three aromatic residues tyrosine, Histidine, Phenylalanine and C terminal carboxylate, resulting in a tyrosinate anion [147,148]. In particular, a tyrosine hydroxylate is formed by delocalisation of the negative charge originating from the Phe carboxylate of angiotensin II (Ang II), through CRS, and activates the AT1 receptor triggering blood pressure, vascular dysfunction, and congestive heart failure. The important role of hydroxyl in Ang II was demonstrated by methylation of tyrosine hydroxyl resulting in loss of agonist activity, as shown in Sarmesin analogs which exhibit competitive antagonist activity [149,150].

Elevated blood pressure and hypertension (HT) are conditions associated with many health issues, especially cardiovascular problems such as heart disease, stroke, and heart failure, with cardiovascular disease being the leading cause of mortality worldwide [151]. From 2025 to 2050, cardiovascular disease prevalence is expected to rise by 90.0%, with cardiovascular deaths projected to reach 35.6 million in 2050, up from 20.5 million in 2025 [152,153]. Globally, high blood pressure is responsible for roughly 54% of strokes and 47% of coronary heart disease cases [154].

The major classes of medications for blood pressure control are angiotensin-converting enzyme inhibitors (ACEIs), angiotensin II receptor blockers (ARBs), dihydropyridine calcium channel blockers (CCBs), diuretics (thiazides and thiazide-like diuretics), and beta-blockers. According to the European Society of Cardiology guidelines, ACE, ARBs, CCBs, and diuretics are recommended as first-line treatment [155,156].

### 3.2. Heart Failure

Heart failure (HF) is a clinical syndrome caused by structural and/or functional heart diseases that increase intracardiac pressure and/or impair cardiac output, affecting approximately 1–2% of adults [157,158]. In clinical practice, it is well-known that the RAAS plays a pivotal role in heart failure (HF). Unlike ACE inhibitors, ARBs act downstream by blocking Ang II from attaching to AT1 receptors. This is believed to cause Ang-II to bind more to AT2 receptors, potentially providing greater antifibrotic benefits than ACE inhibitors. However, clinical evidence supporting the effectiveness of ARBs in heart failure patients is less robust compared to that for ACE inhibitors and thus ARBs are recommended in patients who are intolerant to ACEIs or ARNI (sacubitril/valsartan) [159]. ARNI is a combination of ARBs and neprilysin inhibitors and is recommended in patients with HF who remain symptomatic despite the optimal treatment with ACEIs or ARBs. According to the European Society of Cardiology (ESC) 2023 guidelines, ARBs are recommended for patients with Heart Failure with Reduced Ejection Fraction (HFrEF) (Class I) and Heart Failure with Mid-Range Ejection Fraction (HFmrEF) (Class IIa) who are intolerant to ACEIs and ARNI [160].

### 3.3. Chronic Kidney Disease

Globally, in 2021, more than 850 million people were affected by kidney disease, approximately twice the number of people living with diabetes (422 million) and 20 times the global prevalence of cancer (42 million). According to the KDIGO (Kidney Disease: Improving Kidney Outcomes) 2024 guidelines, ARBs and ACE are recommended for people with chronic kidney disease (CKD). Specifically, they are recommended for patients with CKD and severely increased albuminuria (1B), for those with moderately increased albuminuria (2C), and for individuals with moderate-to-severe albuminuria and diabetes (1B) [161]. In summary, from a mechanistic point of view:

- Ang II acting via AT1R activates signaling cascades—MAPK (Mitogen-activated protein kinase)/ERK (Extracellular signal-regulated kinase), JNK (c-Jun N-terminal kinase), STAT (Signal transducer and activator of transcription), NF-κB (Nuclear factor kappa light chain enhancer of activated B cells), and Activator Protein (AP)-1—to drive fibrosis, inflammation, cell proliferation, and proteinuria in CKD. AT2 receptors counter these effects via inhibitory signaling [162];
- Ang II as a renal growth factor, stimulates proliferation of mesangial/tubular cells and fibroblasts, promoting extracellular matrix (ECM) accumulation and Transforming Growth Factor (TGF)-β induction. RAS blockade (ACE inhibitors, AT1R antagonists) prevents proteinuria, fibrosis, and inflammatory infiltration [162]; and
- On podocytes—a key filtration-cell type—Ang II causes cytoskeletal disruption, ROS production, and apoptosis, driving podocytopathy and glomerulosclerosis. RAS blockade protects structurally and functionally [163].

### 3.4. Acute Coronary Syndrome

Cardiovascular disease (CVD) remains the leading cause of death and disability globally and in many cases, acute coronary syndrome (ACS) serves as the initial clinical presentation of CVD [164,165,166]. Secondary prevention following ACS is essential to improve quality of life and reduce both morbidity and mortality. It should be initiated as soon as possible after the initial event. ARBs are recommended in patients with intolerance of ACE inhibitors after ACS with HF symptoms, Left Ventricular Ejection Fraction (LVEF) < 40%, hypertension, and/or CKD [167]. In summary, from a mechanistic point of view:

- Systemic and local RAS activation drives remodeling and worsens outcomes post–MI (myocardial infarction) [168];
- Ang II activates NADPH (Nicotinamide Adenine Dinucleotide Phosphate) oxidase, causing oxidative injury and atherosclerosis [169];
- Ang II, AT1R, and ACE co-localize in plaques, promoting interleukin IL-6 release and instability [170];
- ARBs post-MI upregulate ACE2/Ang (1–7)/MAS (MAS proto-oncogene) and inhibit fibrosis [171]; and
- The ACE2/Ang (1–7)/MAS axis mitigates ischemia–reperfusion injury (IRI) via anti-inflammatory, antioxidant signaling [172].

### 3.5. Alzheimer’s Disease

Alzheimer’s disease (AD) is a well-known progressive form of neuronal cell degeneration, which influences older humans and is estimated to affect 139 million people by 2050 [173]. Although AD is a multifactorial disease and the most prevalent type of dementia among older adults, its main cause is still not fully understood. The prevailing theory for the pathophysiology of Alzheimer’s is the amyloid theory. In this theory, the amyloid precursor protein (APP) is being hydrolyzed by β-secretase and γ-secretase to yield the insoluble amyloid-beta (Aβ) [174]. This is then accumulated in the brain to finally form the Aβ plaques (senile plaques). Elevated levels of Aβ protein are toxic to mature neurons, leading to the shrinking of dendrites and axons, which eventually results in neuronal death. Beta-site amyloid precursor protein-cleaving enzyme 1 (BACE1) inhibitors, designed to decrease Aβ levels, have been tested for many years; however, none have successfully passed clinical trials. In fact, one of them (Lanabecestat) was able to lower cerebrospinal fluid (CSF) Aβ levels by up to 75%. However, on 12 June 2018, phase II/III trials of Lanabecestat were discontinued due to a lack of efficacy. The same happened to many other inhibitors of the same class, as they had very minimal effects on protecting cognitive decline, in some cases worsening it [175,176,177]. Thus, other molecular targets should be determined for the improvement of Alzheimer’s pathology.

The cerebrovascular aspect of Alzheimer’s disease has frequently been overlooked because of the traditional separation between vascular dementia and AD pathology—an outdated distinction that no longer holds [178,179]. Recently, emerging preclinical and clinical evidence has associated the brain renin–angiotensin system (RAS) to AD pathology. Consequently, several elements of the brain RAS—such as angiotensin II type 1 (AT1), angiotensin IV (AT4), and MAS receptors—have been found to be altered in both AD patients and mouse models. Together, the alterations seen in the RAS are believed to play a role in several key neuropathological features of AD, such as neuronal damage, cognitive decline, and vascular problems. Growing evidence has also shown that antihypertensive drugs targeting the RAS—especially angiotensin receptor blockers (ARBs) and angiotensin-converting enzyme inhibitors (ACEIs)—can help delay the onset and progression of Alzheimer’s disease [180,181]. Moreover, these drugs can have a positive effect on vascular dementia [182]. In a clinical study of non-hypertensive individuals with prodromal Alzheimer’s disease, candesartan was found safe and appeared to reduce brain amyloid biomarkers, improve subcortical brain connectivity, and support cognitive function. These results indicate that candesartan could play a significant therapeutic role in Alzheimer’s disease and highlight the need for further research, given the current lack of effective treatment options for this condition [183]. In summary, from a mechanistic point of view:

- Ang II via AT1R increases amyloid-β (Aβ) by upregulating APP mRNA, β-secretase activity, and presenilin expression; it also promotes tau phosphorylation and reactive oxygen species (ROS) generation [184];
- AT1R activation contributes to neuroinflammation, oxidative stress, Aβ accumulation, all implicated in AD pathogenesis [185];
- Overactivation of the Ang II/AT1R axis leads to blood–brain barrier (BBB) disruption, and neurotoxicity [186],
- Brain aging shows an imbalance favoring renin/ACE1/Ang II/AT1R activation, contributing to cognitive decline and neuroinflammation [187];
- Ang II/AT1R-mediated vasoconstriction impairs neurovascular coupling, undermining cerebrovascular function [188]; and
- Hyperactivation of AT1Rs has been shown to induce NADPH oxidase activity that leads to ROS production, thereby prompting oxidative stress, a pathway activated by Aβ in AD [180].

### 3.6. Parkinson’s Disease

Parkinson’s disease (PD) is the most common movement disorder and ranks as the second most widespread neurodegenerative disease. Although Parkinson’s disease (PD) has traditionally been seen mainly as a motor disorder marked by bradykinesia, muscle stiffness, resting tremors, and balance problems, it also includes non-motor symptoms that significantly impact patients’ quality of life. Neuropsychiatric symptoms, such as mood changes, cognitive decline, and psychosis, are the most common among these. Besides reducing quality of life, they also increase the caregiver’s workload and raise the likelihood of institutionalization. Depression is the most common mood disorder, impacting up to 50% of patients as the disease progresses, while anxiety—though frequently occurring alongside depression—has received comparatively less research attention [189]. Evidence suggest that the local nigrostriatal RAAS likely plays a role in regulating dopaminergic neurotransmission, blood flow, and inflammatory responses [190]. The intricate interaction between angiotensin (Ang) and dopamine (DA) relies on the balance of D1, D2, AT1, and AT2 receptors. While the D1 receptor promotes Ang production, the D2 receptor inhibits it. In the same way, AT1 receptors increase DA tone, whereas AT2 receptors decrease it. Notably, PD patients exhibit reduced Ang II binding in the basal ganglia, but it is uncertain if this is a cause or an effect of the neurodegeneration seen in PD. In animal models of PD, neuroprotective effects have been demonstrated with the angiotensin-converting enzyme (ACE) inhibitors captopril and perindopril, as well as the AT1 receptor antagonists losartan, candesartan, and telmisartan. These effects seem to be driven by a decrease in the excessive production of reactive oxygen species (ROS). In a proof-of-concept, randomized, double-blind, crossover study involving PD patients, perindopril enhanced the effects of levodopa without causing dyskinesias. A cohort study in hypertensive patients suggested that ACE inhibitors may reduce the risk of developing PD. The RAS represents a promising target for both symptomatic and neuroprotective therapies in PD. Further research involving PD animal models and patients is needed [190,191,192]. In summary, from a mechanistic point of view:

- Ang II induces dopaminergic neuron apoptosis via NADPH oxidase–mediated ROS [193];
- Overactivation of Ang II/AT1R exacerbates neurodegeneration in PD models [194];
- Brain RAS–dopamine dysregulation promotes neuroinflammation and degeneration [195]; and
- Local RAS in substantia nigra increases vulnerability to degeneration [195].

Certain ARBs possess higher blood–brain barrier (BBB) penetration when compared to others. For instance, losartan, olmesartan, eprosartan, and irbesartan do not cross the BBB, while telmisartan and candesartan are able to cross it. The reason for this is due to their lipophilicity values (logP). Specifically, the logP values are as follows: losartan (1.19) and its active metabolite EXP3174 (−2.45) [196], olmesartan (4.31) [197], eprosartan (3.9) [198], irbesartan (4.56) [199], telmisartan (5.9) [200] and candesartan (6.2) [201]. Thus, it can be deduced that since telmisartan and candesartan have the greater lipophlicity, it is a lot easier for them to cross the blood–brain barrier (BBB). In animal studies, ARBs have been shown to safeguard against impaired cerebral blood flow, neuroinflammation, and neuronal injury. BBB-permeable ARBs may therefore offer particular advantages, as they can penetrate the brain parenchyma to exert direct neuronal effects. In addition to regulating sympathetic activity and hormone production, the brain RAS also influences microglial activation, oxidative stress, cognition, memory, and anxiety-related behavior [202], thus showing salutary effects on cognition and cerebrovascular disease, as well as Alzheimer’s disease (AD) neuropathology. A meta-analysis found that in a large, international, and cognitively intact sample, the use of BBB-crossing ARBs was linked to better memory recall over 3 years of follow-up compared with their non-BBB-crossing counterparts [203].

### 3.7. Anxiety

Recent studies indicate that local RAS circuits in the brain influence cardiovascular regulation, anxiety (AT), depression, and memory consolidation, with disruptions associated with Alzheimer’s, Parkinson’s, and other neurodegenerative disorders. Bordet et al. [204] performed a clinical study where twelve patients were treated with ARBs and 42 with ACE inhibitors (ACEIs). ARB-treated patients had lower State-Trait Anxiety Inventory (STAI) scores than those on ACEIs or drug-free at baseline and during the follow-up. None of the drugs had an impact on depression scores during the study [204]. In summary, from a mechanistic point of view:

- Overactivation of the RAS—particularly through AT1R—drives HPA (Hypothalamic–Pituitary–Adrenal) axis hyperactivity, resulting in anxiety-like behaviors. In contrast, AT1R blockers exert anxiolytic effects by normalizing RAS and HPA activity [205],
- Ang II via AT1R is localized to stress-sensitive brain regions (e.g., hypothalamus) and has been shown to stimulate CRH (Corticotropin-releasing hormone) production, AVP (arginine vasopressin) release, and adrenal catecholamine output, thereby amplifying stress responses [206]; and
- Stress-induced high Ang II levels cause anxiogenesis via AT1R, and AT2R appears to mediate anxiolytic effects. This suggests that AT2R agonism may counterbalance AT1R-driven anxiety [207].

### 3.8. Cancer Glioma

In cancer therapy, Konain et al. [208] found that AT1 antagonists showcase strong anticancer potential for glioma. Glioma (GC) is the most common and aggressive type of brain tumor and ranks among the deadliest forms of cancer. Among many abnormally expressed genes, the AT1 receptor is reported to be increased in glioma and linked to aggressive tumor characteristics and disease progression. Thus, the research team performed docking studies to eleven FDA approved ARBs and the drug with the highest docking score was selected for in vitro experimentation. In vitro growth inhibitory assays on patient-derived glioma cell lines showed that telmisartan, at a concentration of 45 ± 0.06 μM, could suppress 50% of the malignant glioma U87 cell population, while PCR (Polymerase Chain Reaction) assays showed that AT1 expression in the untreated sample was high reinforcing the role which exhibits the AT1 receptors on glioma. The results of this study indicate that telmisartan effectively inhibits AT1 expression in glioma cell lines [208]. A phase I clinical trial, where patients with glioblastoma are treated with a combination of RAS modulators, showed that the treatment was well tolerated with low side-effects, preserves the quality of life and performance status of the patients, and may lengthen survival time [209]. Candesartan and other sartans manifest their activity through warhead anionic tetrazolates or carboxylates which bind and block the activity of positive arginines that trigger SARS-CoV-2 [210] and other arginine-based diseases such as glioblastoma [211,212]. ARBs and, in particular, bisartans bearing two tetrazoles, are promising candidates to be explored for the treatment of glioblastoma. In summary, from a mechanistic point of view:

- Glioblastoma cells express renin, angiotensinogen, renin receptor, ACE, AT1R, AT2R, and renin inhibition induces apoptosis [213];
- Losartan decreases glioma growth, angiogenic factors, increases apoptosis [214];
- In a rat glioblastoma (C6 glioma) model, Ang (1–7) inhibited the JNK (c-Jun N-terminal kinase) pathway, which is activated by GBM and known to disrupt tight junction proteins. Blocking JNK preserved endothelial junction integrity, reduced vascular leak, and limited tumor-induced edema [215].

### 3.9. Pathogenic Inflammation

Pathogenic inflammation (PI) is typically triggered by infections, which stimulate the release of pro-inflammatory mediators like tumor necrosis factor alpha (TNF-α), interleukin-6 (IL-6), and nitric oxide. While infection is the primary cause of inflammation, it has also been shown that danger signals originating from the host or the environment can provoke sterile inflammation. Inflammasomes are protein complexes within the cytosol that detect pathogen infections and various sterile danger signals, triggering the onset of inflammatory diseases and inflammation-associated diseases such as cardiovascular disease, diabetes, and obesity. Of the known inflammasomes, the NLRP3 (NOD-like receptor family pyrin domain containing 3) inflammasome reacts not only to pathogen infections but also to sterile danger signals originating from the host or environment. Therefore, targeting the NLRP3 inflammasome has become a highly desirable drug target to treat a wide range of human diseases. Candesartan is an angiotensin II receptor antagonist widely used as a blood pressure-lowering drug. Lin et al. [216] showed that candesartan effectively suppressed the NLRP3 inflammasome and pyroptosis in macrophages. Their mechanistic study found that candesartan reduced the expression of NLRP3 and proIL-1β by inhibiting NF-κB activation and decreasing phosphorylation of ERK1/2 and JNK1/2. Additionally, it lessened mitochondrial damage and blocked NLRP3 inflammasome assembly by preventing NLRP3 from binding to protein kinase R (PKR), NIMA-related kinase 7 (NEK7), and apoptosis-associated speck-like protein containing a CARD (ASC). Furthermore, candesartan partially inhibited IL-1β secretion by promoting autophagy. These findings suggest that candesartan possesses broad anti-inflammatory properties and could potentially be repurposed to treat inflammatory diseases or complications related to NLRP3 [216]. A meta-analysis of randomized controlled trials found that ARBs significantly reduced levels of inflammatory markers such as C-reactive protein (CRP), IL-6, and TNF-α [217]. The relationship between angiotensin receptor blockers (ARBs), experimental auto-immune encephalomyelitis (EAE), and multiple sclerosis (MS) has been increasingly explored in recent research. ARBs exhibit anti-inflammatory, neuroprotective, and immunomodulatory properties, making them candidates for repurposing in neuroinflammatory conditions like multiple sclerosis [218,219]. Myelin Basic Protein (MBP) antigens, linear and cyclic, known to contain many arginines, have been extensively studied by us to investigate cytokine secretions in peripheral blood mononuclear cells (PBMC) in multiple sclerosis patients [220] and structural requirements for the binding of MBP peptides to the Major Histocompatibility Complex Class II molecule (MHC II) [221]. The abundant content of basic arginines in MBP (total 19 residues) makes it an attractive peptide to investigate the effects of ARBs and bisartans containing warhead anionic tetrazoles that strongly bind to arginines [222].

### 3.10. Candidosis

Candidosis (CS) is a common opportunistic infection that can present in various clinical forms, including localized infections in the mouth. Medications targeting the renin-angiotensin system inhibit secreted aspartic proteases produced by *Candida albicans*. Preclinical studies by Lara et al. [223] showed that these antihypertensive medications could be repurposed to disrupt the metabolism and formation of *Candida* biofilms, which are commonly linked to clinical candidosis, including oral localized forms like denture stomatitis. Biofilms were exposed to losartan or aliskiren (for comparison) for 24 h. Both drugs decreased fungal viability at all concentrations [223]. No human trials have yet been performed for the treatment of Candidosis.

### 3.11. Fibrosis

Research also offers strong support for the anti-fibrotic effects triggered by activating the AT2 receptor of the RAAS. Stimulation of the AT2 receptor, like when the AT1 receptor is inhibited, has been shown to prevent fibrosis (FS) development in organs such as the lungs, heart, blood vessels, kidneys, pancreas, and skin. In the lungs, AT2 receptor activation even reversed established fibrosis [180,213,224]. From a mechanistic point of view:

- Ang II acts through AT1R leading to TGF-β/Smad (Suppressor of Mothers against Decapentaplegic) activation, ROS, inflammation [225];
- Ang (1–7), acting through the MAS receptor, inhibits fibrosis, reduces inflammation, restores tissue integrity [226]; and
- In liver fibrosis, AT2R is upregulated and exerts antifibrotic effects by inhibiting the IRE1α-XBP1 (Inositol-Requiring Enzyme 1 alpha-X-Box Binding Protein 1) pathway [227].

There are ongoing clinical trials to assess the efficacy of ARBs in reducing fibrosis in conditions such as sickle cell disease (NCT05012631), aortic stenosis (NCT04913870), and acute kidney injury (AKI) (NCT05272878). The effect of angiotensin-blocking agents on liver fibrosis in patients with hepatitis C has also been evaluated by Corey et al. [228]. Patients with hepatitis C and hypertension exhibit greater fibrosis compared to those without hypertension. Among hypertensive patients, those treated with angiotensin-blocking agents showed reduced fibrosis relative to untreated patients. These findings suggest that hypertension, potentially via the renin–angiotensin system, contributes to fibrosis development and indicate a beneficial role for angiotensin II blockade in hepatitis C–related fibrosis [228].

### 3.12. Tissue Fibrosis in Systemic Sclerosis

Tissue fibrosis in systemic sclerosis (TFSSc) results from an excessive buildup of extracellular matrix components produced by fibroblasts in skin lesions. Angiotensin II, a vasoconstrictor peptide, is known to promote fibrosis by stimulating extracellular matrix production. Kawaguchi et al. [229] confirmed this fact and found that abnormal production of Ang II may contribute to tissue fibrosis by causing excessive extracellular matrix production in SSc dermal fibroblasts. This implies that targeting the AT1 receptor with antagonists could offer a new approach for treating tissue fibrosis in SSc patients [229]. A clinical study by Dziadzio et al. [230] was conducted to compare the efficacy and tolerability of losartan, with nifedipine for the treatment of primary and secondary Raynaud’s phenomenon (RP). The tolerability of short-term treatment of RP with losartan was confirmed, and the data suggested its clinical benefit. Further evaluation of this drug as a long-term treatment for SSc-associated RP should be considered, since it may have additional disease-modifying potential [230].

### 3.13. Diabetic Peripheral Neuropathy

Moreover, Iwane et al. [231] conducted clinical and preclinical studies to test whether pharmacological inhibition of the angiotensin system would prevent diabetic peripheral neuropathy (DPN) accompanying type 2 diabetes mellitus (T2DM). In the clinical study, the enrolled 7464 patients were divided into three groups receiving ACEIs, ARBs and the others (non-ACEI, non-ARB antihypertensives). Bonferroni’s test indicated significantly later DPN development in the ARB and ACEI groups than the other groups (receiving non-ACEIs and non-ARBs antihypertensives). The results suggests that pharmacological inhibition of the angiotensin system is beneficial to prevent DPN accompanying T2DM [231]. From a mechanistic point of view:

- Spinal Ang II/AT1R signaling drives neuropathic pain via p38 MAPK; blocked by losartan [232];
- ACE inhibition in diabetic rats prevents nerve dysfunction and promotes endoneurial angiogenesis [233]; and
- Ang II–driven ROS via NADPH oxidase confirms broader neurotoxicity of Ang II/AT1R pathway [234].

### 3.14. Inflammatory Bowel Diseases

Inflammatory bowel diseases (IBDs), such as Crohn’s disease (CD) and ulcerative colitis (UC), are long-lasting conditions affecting the gastrointestinal tract, characterized by repeated episodes of inflammation. Current treatments for IBD are not curative and fall short in areas such as preventing fibrosis. Medications targeting the renin–angiotensin system (RAS) not only lower blood pressure but also have anti-inflammatory and antifibrotic effects, making them a cost-effective option for managing inflammation and fibrosis in the gut. While RAS inhibitors have shown promise in preventing and easing colitis in preclinical studies, evidence from human trials remains limited. According to Salmenkari et al. [235] retrospective studies of IBD patients treated with ACEIs or ARBs have shown encouraging results, including milder disease progression, fewer hospitalizations, and reduced corticosteroid use. However, prospective studies are necessary to confirm the effectiveness of these promising drugs in treating IBD [235]. Mechanistically:

- Ang II activates the local RAS leading to the activation of JAK2/STAT1/3, elevated TH1/TH17 (T helper 17) T-cell responses, and IEC apoptosis [236];
- Ang (1–7)/MASR reduces signaling [p38/ERK/Akt (Protein kinase B)] [237].

Jacobs et al. [238] performed a clinical trial where rates of IBD-related hospitalizations, surgeries, and corticosteroid use were retrospectively assessed in two groups. In the first, 111 IBD patients on an ACEI or ARB were compared 1:1 with matched nonusers based on sex, age, diagnosis, disease location, and hypertension status. In the second, outcomes in 130 IBD patients were compared before and during ACEI/ARB treatment. The results indicated that IBD patients treated with ACEIs or ARBs experienced fewer hospitalizations, surgeries, and corticosteroid courses compared to matched controls. However, no differences in outcomes were observed when patients were compared to their own status prior to ACEI/ARB therapy [238].

### 3.15. Marfan Syndrome

Marfan syndrome (MS) is a genetic disorder typically caused by harmful mutations in the fibrillin-1 (FBN1) gene, leading to gradual enlargement of the aortic root. If left untreated, this aortic dilation can result in life-threatening aortic dissection, occasionally occurring in early adulthood. Based on a meta-analysis by Pitcher et al. [239], in individuals with Marfan syndrome who had not undergone aortic surgery, ARBs reduced the rate of aortic root Z score enlargement by roughly half, even in those also taking beta-blockers. The impact of beta-blockers was comparable to that of ARBs. Assuming additive effects, starting combination therapy with both ARBs and beta-blockers at diagnosis could further reduce the rate of aortic enlargement compared to either treatment alone. If sustained over several years, this approach is expected to delay the need for aortic surgery [239]. From a mechanistical perspective:

- AT1R blockade (losartan) in MS mice prevents aneurysm, reverses pathology via TGF-β/Smad suppression [240];
- AT2R plays a pivotal role for full therapeutic effect; required for ERK inhibition [241];
- Losartan restored proper muscle regeneration in fibrillin-1–deficient mice by antagonizing TGF-β. This demonstrates that AT1R blockade alleviates systemic manifestations of MS (e.g., myopathy, lung architecture defects), not only vascular issues [242]; and
- Ang II/AT1R signaling activates ERK1/2 pathways and TGF-β/Smad, driving extracellular matrix degradation and aneurysm formation [243].

### 3.16. SARS-CoV-2

Furthermore, ARBs and ACEIs have been reported to protect hypertensive patients infected with SARS-CoV-2. Renin–angiotensin system (RAS) inhibitors decrease excess angiotensin II levels while increasing antagonist heptapeptides like alamandine and aspamandine, which help counteract angiotensin II and promote homeostasis and vasodilation. Comprehensive studies have shown that ARBs influence the renin–angiotensin system (RAS) by increasing the levels of the ACE2 enzyme more than other hypertension medications. This is especially significant because ACE2 serves as the entry point for SARS-CoV-2 in the nasopharynx, lungs, and heart cells. Their size, polarity, charge, and receptor selectivity make these drugs well-suited for maintaining homeostasis, suggesting they could be promising therapeutic agents against SARS-CoV-2 infection. ACE2 enzyme, which transforms harmful Ang II into the beneficial peptides Ang (1–7) and alamandine, helps to maintain balance while simultaneously preventing SARS-CoV-2 from entering through ACE2 [244]. A phase III clinical trial with the code name “CLARITY” concluded that no evidence of benefit, based on disease severity score, was found for treatment with angiotensin receptor blockers in patients with mild disease, not requiring oxygen, administered orally 40 mg/day of telmisartan [245]. A seamless phase I and II study, infusing angiotensin (1–7) intravenously in COVID-19 patients admitted to the ICU (Intensive Care Unit) with severe pneumonia at 10 mcg/Kg daily is safe. In the Phase II intention-to-treat analysis, there was no significant difference in oxygen-free days (OFD) between groups. However, phase II was terminated prematurely due to low recruitment rates and funding shortages, limiting the interpretation of these data [246]. Further, studies need to be performed in order to properly assess the therapeutic potential of sartan derivatives.

### 3.17. Rheumatoid Arthritis

Rheumatoid arthritis (RA) and osteoarthritis (OA) represent the two primary types of inflammatory arthritis. Although RA and OA have distinct mechanisms of development, they share certain similarities. In both cases, ongoing inflammation causes gradual damage to the joints. The renin–angiotensin system (RAS) plays a role in the development of both RA and OA [247].

RA is a significant condition that impacts joints by increasing inflammation and leading to periarticular osteopenia. Due to the numerous side effects associated with current RA treatments, finding alternative therapies has become a crucial area of research. A local functional RAAS has been identified in various organs and tissues, such as chondrocytes, synovial fluid, and synovial tissue. ACE and renin concentrations were also higher at the synovial fluid in RA patients. The generated Ang II increases pro-inflammatory cytokines like IL-1, IL-6, and TNF-α, which may play a role in the development of RA. Both experimental and clinical research indicate that RAS inhibitors—especially ARBs, as well as ACE inhibitors and renin inhibitors—play a role in RA by primarily targeting inflammation and oxidative stress [247].

### 3.18. Osteoarthritis

Osteoarthritis (OA) is a painful joint condition characterized by the gradual breakdown of cartilage, resulting in discomfort and reduced movement. Existing diagnostic techniques and the absence of treatments that alter disease progression emphasize the urgent need for new management approaches. Recent studies indicate that the RAAS, especially the effects of Ang II on AT1 and AT2 receptors in synovial tissue, could be crucial in the development of osteoarthritis and rheumatoid arthritis. Excessive activation of AT1R is associated with diseases like hypertension and cardiovascular fibrosis, which have similarities to osteoarthritis. In joint tissues—such as cartilage and synovium—AT1R stimulation by Ang II or inflammatory cytokines like IL-1β triggers the release of pro-inflammatory substances and matrix metalloproteinases (MMPs), speeding up cartilage degradation and worsening joint injury. RAS modulators, including ARBs and ACEIs, are being investigated as possible treatments for osteoarthritis. Kaur et al. [248] found that inhibition of the AT1 receptor shows promise in reducing IL-1β-driven inflammation, extracellular matrix (ECM) breakdown, and chondrocyte death in osteoarthritis, while also promoting ECM production, autophagy, and protection of cartilage cells. These effects are regulated by key transcription factors such as STAT3, NF-κB, MAPK, vascular endothelial growth factor (VEGF), and Caspase 3. The results reveal not only the cartilage-protecting benefits of these drugs but also clarify how they reduce inflammation and support chondrocyte survival, providing valuable understanding for potential treatment options [248]. No clinical trials have been performed with regard to treating OA with sartans. One clinical study assessing the antihypertensive drug-associated adverse events in osteoarthritis in patients with OA and hypertension found that valsartan had strong osteoarthritis adverse reaction signals among the three ARBs, namely, irbesartan, cloxartan, and valsartan [249].

### 3.19. Opioid Addiction

Drug use is a continually rising problem in developed countries, with a representative example being the US where more than 23 million adults struggle with addiction [250]. In 2017, the US Department of Health and Human Services declared the opioid problem as an epidemic, which accounted for more than 42,000 deaths in 2016 [251]. Opiates are much more addictive than other kinds of substances, which poses an even bigger threat in society. In an attempt to tackle opiate addiction, based on the principles of drug repositioning, Ridgway et al. [252] utilized computational chemistry tools to unravel crossover binding patterns of diverse ligands to angiotensin, alpha-adrenergic, and opioid receptors. Their work was based on recent bioassay studies which were in agreement with the high (computationally predicted) binding affinities of angiotensin receptor blockers (ARBs) at α-adrenergic receptors (αARs) in isolated smooth muscle. Via the use of docking and molecular dynamics (MD) simulations, they explored the affinities and stabilities of selected non-peptide ligands (of which and sartans) on several G protein-coupled receptors GPCRs, including α1AR, α2AR, and µ-(µOR) and ժ-opioid receptors (ժOR). This procedure showed that these ligands preferentially bind to the active site on the cell surface of all three GPCR receptors, with a consistent order of ligand preference, while the blockade they present to αARs and µORs has been confirmed by bioassay studies as well. Moreover, in their results, ARBs which exhibit higher affinities for AT1 receptor also demonstrate higher affinities for µORs and ժORs than opiate ligands, such as fentanyl and naltrexone, showcasing the high number of possibilities these molecules can have in combating opiate addiction. Interestingly, ARBs had, also, a higher affinity for αARs than either alpha agonists (epinephrine and phenylephrine) or inhibitors (prazosin and doxazosin). Finally, tested compounds sartans and bisartans appeared to interfere with the desensitization and/or re-sensitization (tolerance) mechanisms of αARs and µORs, thus proposing their potential role in the treatment of methamphetamine and opioid addiction (OPA) [252]. There are no ongoing clinical trials to study the utilization of ARBs in opioid addiction.

A summary of the diseases, the mechanistic rationale, and the involvement of the RAAS in their development is provided in Table 2.

## 4. Novel Synthetic AT1 Antagonists and Dual Inhibitors

As shown in Table 3, non-peptide molecules with a smaller scaffold than common drugs were synthesized and studied in our laboratories. Thus, (5S)-1-benzylo-5-(1H-imidazol-1ylo-methylo-)-2-pyrrolidinone (**MM1**) was created, matching a simpler synthetic root compared to sartans and a significant antihypertensive activity (71% compared to losartan defined as 100% losartan) when injected to anesthetized rabbits made hypertensive by Ang II infusion [284]. Since these molecules possessed strong inhibitory activity against the AT1 receptor, new paths in drug discovery were opened. **MMK2** and **MMK3** were also synthesized, later possessing 80% and 48% antihypertensive activity relative to losartan at the same experimental conditions. However, the in vitro experiments, using membranes with AT1 receptors and cell cultures, showed that compounds **MM1**, **MMK2**, and **MMK3** exhibited negligible activity compared to the reference drug losartan (IC50 ≈ 10^−9^ M) for the AT1 receptor [285].

Continuing our work, our laboratory designed, synthesized, and evaluated derivatives of losartan in vitro and in vivo, as can be seen by the second group shown in Table 3. Derivative **V-8** exhibited an angiotensin II antagonistic effect in vivo (rabbits) that increased with dosage at 2 and 3.5 µmol, similar to the effects seen with losartan. In vitro binding experiments demonstrated that **V-8** exhibited strong affinity for the AT1 receptor, specifically within the nanomolar range, similar to losartan (IC50 of **V-8**: 53.8 ± 6.4 nM; IC50 of losartan: 16.4 ± 1.6 nM) while it did not bind on the AT2 receptor [286,287]. N-substituted 5-butylimidazole derivatives were also synthesized, from which the compound 5-butyl-1-[[20-(2H-tetrazol-5-yl)biphenyl-4-yl]methyl] imidazole-2-carboxylic acid (**30**) exhibited higher binding affinity (−log IC50 = 8.46) for the AT1 receptor compared to losartan (−log IC50 = 8.25) [288]. Moreover, N,N’-symmetrically bis-substituted butylimidazole analogs have been synthesized and studied. From these analogs, compounds **11** (also named **BV6** and **BisA** in other citations), **12a**, **12b**, and **14** (also named **BisB**) showcased higher antagonistic activity (potency) when compared to losartan (**11**; −logIC50 = 9.46, **12a**; −logIC50 = 9.04, **12b**; −logIC50 = 8.54, **14**; −logIC50 = 8.37 and losartan; −logIC50 = 8.25). Specifically, compound **11** was designed to have most of the pharmacological segments of losartan and an additional biphenyltetrazole moiety, resulting in increased lipophilicity. These compounds are bis-alkylated imidazole sartan derivatives, called “bisartans”, designed to fill a lipophilic cavity that sartans do not accommodate [222,288,289,290,291,292].

Since many diseases are actually multifactorial, we decided to expand our research of AT1 inhibition to other drug targets as well. As such dual inhibitors were designed, synthesized, and studied in silico, in vitro, and in vivo. Characteristic examples are quercetin–losartan hybrids for the treatment of glioblastoma multiforme (GBM). In GBM cells, we showed that this (**Q-L**) hybrid retains the binding potential of losartan to the AT1R (**Q-L** IC50; 140 ± 10 nM, losartan IC50; 10.3 ± 1.1 nM) through competition-binding experiments and simultaneously exhibits ROS inhibition and antioxidant capacity similar to native quercetin. Moreover, it appeared that the hybrid can modify the cell-cycle distribution in GBM cells, causing cell-cycle arrest and triggering cytotoxic effects and inhibits cancer cell proliferation and angiogenesis in primary GBM cultures [293]. Another example is a **DHA–losartan hybrid** used as a potent inhibitor of multiple pathway-induced platelet aggregation. The hybrid demonstrated a broad-spectrum antiplatelet effect by inhibiting platelet aggregation via multiple activation pathways, including P2Y12, PAR-1 (Protease-Activated Receptor-1), PAF (Platelet-Activating Factor), COX-1 (cyclooxygenase-1), and collagen receptors (collagen; losartan IC50; 112.9 μΜ, DHA IC50; 185.6 μΜ and **DHA–losartan hybrid** IC50; 249.1 μΜ) [294]. The synthetic routes for AT1 antagonists and more specifically sartan derivatives are established and reviewed by our laboratories and specifically Georgiou et al. [295].

We have also scanned compound databases like ChEMBL15 and discovered a lot of molecules that could possibly be used for AT1 inhibition. All analogs bound to the AT1 receptor in a dose-dependent manner, showing significant binding affinities (−log IC50). Specifically, the −log IC50 values for compounds **1**, **2**, **3**, and **4** (Table 3) were 5.66 ± 0.14, 5.68 ± 0.26, 5.59 ± 0.33, and 6.70 ± 0.19, respectively. Notably, compound **4** exhibited a binding affinity to the AT1 receptor that was approximately 10 times higher than the other compounds and closer to that of losartan, which had a value of 8.49 ± 0.18.73 [296]. All the aforementioned molecules are shown in Table 3.

Based on the bibliographic search we conducted, there are no instances of synthesized small molecules with a small non-peptide scaffold to be comparable to compounds **MM1**, **MMK2**, and **MMK3** as showcased in Table 3. Nevertheless, different scientific groups have given the name “small non-peptide” to their sartan derivatives, and we refer to some of them in Table 4. Below, we showcase representative examples of different sartan derivatives.

**KRH-594** (Table 4), synthesized by Kissei Pharmaceutical Co., Ltd. (Hotaka, Japan) [297,298], is another instance of a non-peptide molecule exhibiting potent pharmacodynamic properties for the inhibition of the AT1 receptor. In particular, the compound potently displaced specific binding of [^125^I]-Ang II at the AT1 receptor with a Ki = 0.39 ± 0.08 nM (*n* = 4). When compared to losartan and its metabolite EXP3174 with Ki values of 14 ± 3.0 nM (*n* = 4) and 0.79 ± 0.18 nM (*n* = 3), respectively, its potency becomes evident ( ~ 36-fold more potent as an AT1 antagonist than losartan and twofold more potent than EXP3174). On the other hand, it did not show the same effects for the AT2 receptor in bovine cerebellar membranes (Ki > 10 μM) (>25,000-fold higher affinity for AT1). The strong specificity of **KRH-594** for the AT1 receptor subtype was further confirmed by its lack of binding affinity to various other receptors and enzymes at a concentration of 10 μM. All these findings demonstrate that **KRH-594** is a potent and highly selective AT1 receptor antagonist [299]. Interestingly, while this compound has a very promising pharmacologic profile according to preclinical studies, no further attempt was made to evaluate this compound in clinical trials to this date.

Another compound worth mentioning is **KT3–671** (now also known as **KD3-671**) (Table 4) synthesized by Mochizuki et al. [300], as a non-peptide molecule for the antagonism of the AT1 receptor. **KT3–671** displaced specific binding of [^125^I]Sar^1^ Ile^8^-Ang II to AT1 receptor with a Ki value of 0.71 ± 0.14 nM in rat liver membranes, but had no affinity for AT2 receptor in bovine cerebellar membranes (Ki > 10 μM). When compared with losartan (DuP 753) and EXP3174, which displaced the specific binding of the radioligand to AT1 receptor, with Ki values of 5.02 ± 1.63 and 0.32 ± 0.06 nM, respectively, it shows a strong affinity for this receptor. This compound went also to clinical trials where in a phase II randomized double-blind study of patients with mild to moderate essential hypertension, **KT3-671** (20–80 mg) demonstrated a shallow dose–response relationship for reductions in sitting trough blood pressure (BP). Significant effects were observed only for sitting office diastolic BP compared with placebo, while reductions in ambulatory blood pressure (ABP) were more pronounced than those in office trough BP [300]. Another study aimed to evaluate the antihypertensive efficacy of once-daily **KT3-671** doses of up to 160 mg (40, 80, and 160 mg) compared with placebo in patients with mild to moderate uncomplicated essential hypertension. Only the 40 mg dose showed a statistically significant difference from the placebo group for the primary efficacy measure. The researchers concluded that, at suitable doses, **KT3-671** could serve as an effective once-daily antihypertensive agent. It was well tolerated across all three tested doses, consistent with the characteristic of AT1 receptor blockers that adverse effects are not dose-dependent [301].

Many derivatives of losartan have been synthesized throughout the years, with the intention of improving its affinity to the AT1 receptor and the lives of patients. Han et al. [302] developed novel angiotensin II receptor type 1 (AT1) blockers bearing 6-substituted carbamoyl benzimidazoles with a chiral center and tried to understand its pharmacodynamic properties. Blood-pressure screening in spontaneously hypertensive rats revealed that the most pronounced activity, compared to losartan (IC_50_ = 28.6 ± 2.0 nM), was observed with compound **8R** (IC_50_ = 1.1 ± 0.5 nM). Compound **8R** (Table 4) was identified as a promising candidate due to its strong antihypertensive efficacy and relatively low toxicity, as evidenced by plasma analyses, toxicology studies, and chronic oral testing. Docking studies further revealed that **8R** forms multiple robust interactions with the active sites of the theoretical AT1 receptor model [302].

Zhu et al. [303] designed, synthesized and evaluated in vitro and in vivo, 6-substituted benzimidazole with 1, 4-disubsituted or 1, 5-disubsituted indole derivatives as novel angiotensin II receptor antagonists. Biological evaluation in spontaneously hypertensive rats demonstrated that 2-[4-[[2-n-propyl-4-methyl-6-(1-methylbenzimidazol-2-yl)benzimidazole-1-yl]methyl]-1H-indol-1-yl]benzoic acid; compound **1c** (IC50 = 0.36 ± 0.18 nM, Ki = 0.23 ± 0.17 nM) (Table 4) produced a significant, dose-dependent reduction in mean blood pressure (MBP). Oral administration resulted in maximal decreases of 53 mmHg at 5 mg/kg and 64 mmHg at 10 mg/kg, with the antihypertensive effect persisting for over 24 h—surpassing the efficacy of both losartan (IC50 = 20.09 ± 0.11 nM, Ki = 13.06 ± 0.07 nM) and telmisartan (IC50 = 3.80 ± 0.22 nM, Ki = 2.75 ± 0.17 nM). Acute toxicity studies further indicated that 1c exhibited low toxicity, with no notable weight changes or adverse reactions observed, making it an effective and durable anti-hypertension drug candidate, deserving further investigation for therapeutic application [303].

5-oxo-1,2,4-oxadiazole derivatives with 1, 4-disubsituted or 1, 5-disubsituted indole group were, also, designed, synthesized, and pharmacologically evaluated by Zhu. et al. [304]. These derivatives exhibited strong affinities for the AT1 receptor, comparable to losartan (IC50 = 10.51 ± 2.19 nM, Ki = 7.61 ± 1.59 nM), but they were not as potent as irbesartan (IC50 = 1.30 ± 0.06 nM, Ki = 0.94 ± 0.04 nM). The methyl ester containing a 1,4-disubstituted indole group, compound **1** (IC50 = 5.01 ± 1.67 nM, Ki = 3.63 ± 1.21 nM) (Table 4), demonstrated potent antihypertensive activity in spontaneously hypertensive rats (SHRs). Following oral administration at 10 mg/kg, it reduced mean blood pressure (MBP) by 30 mmHg, surpassing the effect of irbesartan, with the antihypertensive response persisting for over 24 h. Compound **1** had low acute toxicity; however, hyperthyroidism appeared 6 h later after administration in other groups, and some mice died. There were no significant changes in the weight of the surviving mice after 2 weeks of observation and no obvious untoward reactions appeared [304].

A series of novel oxadiazole derivatives were designed, synthesized, and evaluated for their pharmacological effects by Qu et al. [305]. These compounds exhibited strong affinity for the AT1 receptor and produced significant blood pressure reductions in spontaneously hypertensive rats at nanomolar concentrations. Notably, compounds **IV1** (Table 4) with an IC50 value of 7.7 ± 1.2 nM and Ki = 5.5 ± 0.6 nM and **IV2** (Table 4) with IC50 = 8.0 ± 0.5 nM and Ki = 5.8 ± 0.4 nM proved especially effective, showing equal or greater potency than losartan (IC50 = 14.6 ± 1.6 nM, Ki = 10.5 ± 1.2 nM), highlighting their potential as candidates for antihypertensive drug development [305].

Tang et al. [306] designed and synthesized (2-(4-((2-amyl-5-nitro-1H-benzo[d]-imidazol-1-yl) methyl)-1H-indol-1-yl) tetrazole); compound **1a** (Table 4). The compound **1a** had a higher affinity to bind with AT1 receptor (**1a**: IC 50 = 4.05 ± 2.11 nM, Ki = 2.93 ± 1.53 nM) compared to losartan (IC50 = 12.23 ± 3.42 nM, Ki = 8.86 ± 2.49 nM). It was prepared and orally administered to spontaneous hypertensive rats to study the antihypertensive effects. The maximum reduction in blood pressure reached 50 mmHg after dosing compound **1a** for 5 h (dose of 10 mg/kg). However, at the same dose, losartan reached the maximum hypotensive value at 45 mmHg in 3 h and antihypertensive activity in 12 h. Compound **1a** demonstrated greater efficacy in reducing blood pressure at the same dose, with improved stability and a more sustained effect [306].

Zhu et al. [307] designed, synthesized, and evaluated 5-nitro benzimidazole in vitro and in vivo with 1,4-disubsituted or 1,5-disubsituted indole derivatives as novel angiotensin II receptor antagonists. Radioligand-binding assays showed that 2-(4-((2-butyl-5-nitro-1H-benzo[d]imidazol-1-yl)methyl)-1H-indol-1-yl)benzoic acid, compound **3** (Table 4), displayed a high affinity for the angiotensin II type 1 receptor with IC50 value of 1.03 ± 0.26 nM and Ki value of 0.97 ± 0.43 nM (higher compared to losartan; IC50 = 3.54 ± 0.34 nM, Ki = 2.53 ± 1.12). The biological evaluation on spontaneously hypertensive rats and renal hypertensive rats showed that **3** could cause a significant decrease on MBP in a dose-dependent manner, whose maximal response lowered 30 mmHg of MBP at 5 mg/kg and 41 mmHg of MBP at 10 mg/kg after oral administration, and the significant antihypertensive effect lasted beyond 24 h, which is better than losartan. Taken together, **3** could be considered as an effective and durable anti-hypertension drug candidate. These encouraging results deserve further investigation towards its use for therapeutic benefit [307].

**Table 3:** Presentation of the key compounds with significant biological activity that were designed, synthesized, and studied computationally and pharmacologically by our research team and collaborators over the years, grouped according to the methodology used to design them. Four major groups are outlined. Group A, small non-peptide molecules; Group B, sartan derivatives; Group C, hybrid molecules; and Group D, databases.

Structures of Bioactive Compounds	Biological Evaluation
*Group A: small non-peptide molecules*	
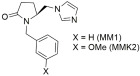	Significant antihypertensive activity (**MM1**: 71% and **MMK2**: 80% when compared to losartan defined as 100% losartan) when injected to anesthetized rabbits made hypertensive by Ang II infusion. In vitro experiments showed that compounds **MM1** and **MMK2** exhibited negligible activity compared to the reference drug losartan [284,285].
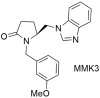	Antihypertensive activity (**MMK3**: 48% when compared to losartan defined as 100% losartan) when injected to anesthetized rabbits made hypertensive by Ang II infusion. In vitro experiments showed that compound **MMK3** exhibited negligible activity compared to the reference drug losartan [285].
*Group B: sartan derivatives*	
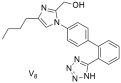	In vitro binding studies; **V8** exhibits affinity in the nanomolar range similar to losartan for the AT1 receptor (**V8**: IC50 = 53.8 ± 6.4 nM and losartan; IC50 = 16.4 ± 1.6 nM). **V8** is a selective AT1 antagonist [286,287].
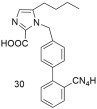	Higher binding affinity of compound **30** compared to losartan (**30**: −logIC50 = 8.46; and losartan: −logIC50 = 8.25). Importance of carboxy group at the C-2 position [288].
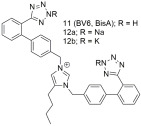	Compounds **11** (also named BV6 or BisA), **12a** and **12b** showcase higher antagonistic activity (potency) when compared to losartan (**11**: −logIC50 = 9.46; **12a**: −logIC50 = 9.04; **12b**: −logIC50 = 8.54; and losartan: −logIC50 = 8.25). Compound’s **11** elevated docking score for the AT1 receptor is due to a greater number of hydrophobic interactions compared to losartan [222,288,289,290,291,292].
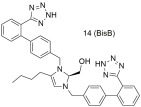	Compound **14** (also named **BisB**) showcases higher antagonistic activity (potency) when compared to losartan (**14**: −logIC50 = 8.37; and losartan: −logIC50 = 8.25) [222,288,289,290,291,292].
*Group C: hybrid molecules*	
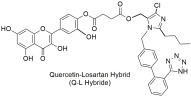	The quercetin–losartan (**Q-L**) hybrid retains the binding potential of losartan to the AT1R (**Q-L** IC50: 140 ± 10 nM; losartan IC50: 10.3 ± 1.1 nM), exhibits ROS inhibition and antioxidant capacity similar to native quercetin, modifies the cell-cycle distribution in GBM cells, and inhibits cancer cell proliferation and angiogenesis in primary GBM cultures [293].
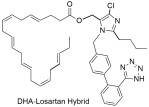	**DHA–losartan**; potent inhibitor of multiple pathway-induced platelet aggregation, like P2Y12, PAR-1 (Protease-Activated Receptor-1), PAF (Platelet-Activating Factor), COX-1 (cyclooxygenase-1), and collagen receptors (collagen; losartan IC50: 112.9 μΜ; DHA IC50: 185.6 μΜ; and **DHA–losartan hybrid** IC50: 249.1 μΜ) [294].
*Group D: databases*	
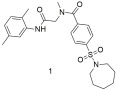	Compound **1** shows good binding affinity for the AT1 receptor but not better than losartan (**1**: −logIC50 = 5.66 ± 0.14; and losartan: −logIC50 = 8.49 ± 0.18) [296].
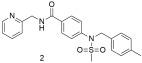	Compound **2** shows good binding affinity for the AT1 receptor but no better than losartan (**2**: −logIC50 = 5.68 ± 0.26; and losartan: −logIC50 = 8.49 ± 0.18) [296].
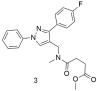	Compound **3** shows the worst binding affinity for the AT1 receptor compared to **1** and **2** and no better than losartan (**3**: −logIC50 = 5.59 ± 0.33; and losartan: −logIC50 = 8.49 ± 0.18) [296].
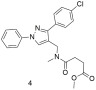	Compound **4** has 10-fold higher binding affinity for the AT1 receptor compared to **1**, **2**, and **3** but is not better than losartan (**4**: −logIC50 = 6.70 ± 0.19; and losartan: −logIC50 = 8.49 ± 0.18) [296].

**Table 4:** Presentation of selected compounds with significant biological activity that were designed, synthesized, and studied pharmacologically by different research groups over the years, for comparative purposes.

Structures of Bioactive Compounds	Biological Evaluation
*Group A; Sartan Derivatives*	
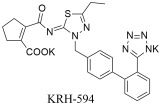	In vitro binding studies; higher affinity of **KRH-594** when compared to losartan and its active metabolite EXP3174 for the AT1 receptor [**KRH-594**: Ki = 0.39 ± 0.08 nM (*n* = 4), losartan; Ki = 14 ± 3.0 nM (*n* = 4) and Ki = 0.79 ± 0.18 nM (*n* = 3)] [299].
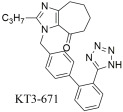	In vitro binding studies; higher affinity of **KT3–671** when compared to losartan and its active metabolite EXP3174 for the AT1 receptor [**KT3–671**: Ki = 0.71 ± 0.14 nM; losartan (DuP 753): Ki = 5.02 ± 1.63 nM (*n* = 4); and EXP3174: Ki = 0.32 ± 0.06] [301]. Strong affinity for this receptor in rat liver membranes.
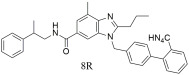	In vitro binding studies; higher activity of **8R** when compared to losartan for the AT1 receptor (**8R**: IC50 = 1.1 ± 0.5 nM and losartan: IC50 = 28.6 ± 2.0 nM). Promising candidate due to its strong antihypertensive efficacy and relatively low toxicity, as evidenced by plasma analyses, toxicology studies, and chronic oral testing [302].
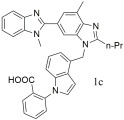	Oral administration of compound **1c** (IC50 = 0.36 ± 0.18 nM, Ki = 0.23 ± 0.17 nM) resulted in maximal decreases of 53 mmHg at 5 mg/kg and 64 mmHg at 10 mg/kg, with the antihypertensive effect persisting for over 24 h—surpassing the efficacy of both losartan (IC50 = 20.09 ± 0.11 nM, Ki = 13.06 ± 0.07 nM) and telmisartan (IC50 = 3.80 ± 0.22 nM, Ki = 2.75 ± 0.17 nM) [303].
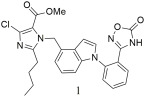	Compound **1** (IC50 = 5.01 ± 1.67 nM, Ki = 3.63 ± 1.21 nM) shows good binding affinity for the AT1 receptor, comparable to losartan (IC50 = 10.51 ± 2.19 nM, Ki = 7.61 ± 1.59 nM), but weaker than irbesartan (IC50 = 1.30 ± 0.06 nM, Ki = 0.94 ± 0.04 nM). It reduced MBP by 30 mmHg, surpassing the effect of irbesartan, and had low acute toxicity [304].
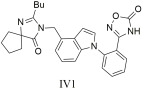	Compound **IV1** (IC50 = 7.7 ± 1.2 nM, Ki = 5.5 ± 0.6 nM) proved especially effective, showing greater potency than losartan (IC50 = 14.6 ± 1.6 nM, Ki = 10.5 ± 1.2 nM), highlighting its potential as a drug candidate [305].
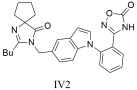	Compound **IV2** with IC50 = 8.0 ± 0.5 nM and Ki = 5.8 ± 0.4 nM showed greater potency than losartan (IC50 = 14.6 ± 1.6 nM, Ki = 10.5 ± 1.2 nM), highlighting its potential as a candidate for antihypertensive drug development [305].
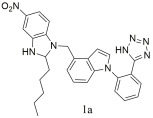	Compound **1a** has a higher affinity to bind with AT1 receptor (**1a**: IC 50 = 4.05 ± 2.11 nM, Ki = 2.93 ± 1.53 nM) compared to losartan: (IC50 = 12.23 ± 3.42 nM, Ki = 8.86 ± 2.49 nM) [306].
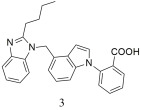	Compound **3**, displayed a high affinity for the angiotensin II type 1 receptor with IC50 value of 1.03 ± 0.26 nM and Ki value of 0.97 ± 0.43 nM (higher compared to losartan; IC50 = 3.54 ± 0.34 nM, Ki = 2.53 ± 1.12) [307].

**Figure 2:**
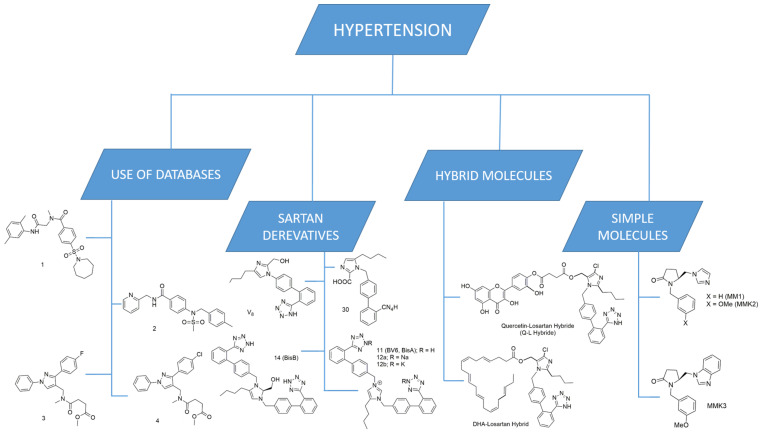
A schematic representation of the compounds designed, synthesized, and studied in silico, in vitro, and in vivo by our laboratory and collaborators over the years. All of them can be separated into 4 major groups: (**1**) molecules discovered and designed with the contribution of databases, (**2**) molecules that are sartan derivatives, (**3**) hybrid molecules, and (**4**) molecules with a simple structure that satisfactorily inhibit AT1 receptors.

The compounds we have synthesized along with our collaborators have been organized into four separate groups and can be seen in Figure 2.

## 5. Bisartans: Second-Generation Non-Peptide Mimetics of Ang II as Pan-Antiviral Drugs

Second-generation ARB bisartans are discovered in our laboratories [222,288,289,290,291,292], in which both imidazole nitrogens are substituted with biphenyltetrazole and exhibit remarkable affinity and strong binding to the catalytic sites of viruses SARS-CoV-2, influenza, and Respiratory Syncytial Viruses, rendering them potential pan-antiviral drugs [308]. This property may derive from the dual interaction of both warhead negative tetrazoles with positive arginines which trigger infections [222,309]. For an analytical presentation of the interactions and the anti-viral effects exerted by our bisartans compared to other drug entities, you can refer to the article by Ridgway et al. [308]. Pioneer research earlier on the design and synthesis of losartan analogs has led to the discovery of a new class of ARBs where the imidazole substituents, i.e., the butyl and hydroxyl methylene groups at positions 2 and 4, respectively, are at reversed positions compared to losartan [286,287,288]. These analogs were the basis for further developing bisalkylated derivatives which symmetrically bear two biphenyl tetrazole groups on the two imidazole nitrogens, called bisartans, with notable properties relevant to hypertension and coronavirus 2019 therapies [222,288,289,290,291,292].

The use of benzimidazole as a scaffold instead of imidazole and bis biphenyl tetrazole alkylation resulted in the development of new bisartans, which exhibited the unique binding affinities due to tetrazole and increased aromaticity [310]. Interaction of aromatic phenyl groups with arginines, as between ARBs and AT1R R167, has been previously reported as a dominant binding factor due to the π-π electron interactions [311,312,313,314]. Arginine is a key amino acid in disease, and arginine blockers, like ARBs or bisartans containing warhead anionic tetrazoles, are emerging as promising pharmaceutics to battle arginine-based viruses and other diseases. The unique and fascinating properties of tetrazole have recently received significant attention in medicinal chemistry for innovative therapies [211,315].

## 6. Conclusions

The renin–angiotensin–aldosterone system (RAAS) plays a crucial role in regulating vascular tone and maintaining fluid balance. In the classical pathway, prorenin is converted into renin in the kidneys. Afterwards, renin converts angiotensinogen into angiotensin I (Ang I) which is metabolized to angiotensin II (Ang II) and binds to angiotensin II type 1 (AT1) receptors, resulting in elevated blood pressure through vasoconstriction, sympathetic activation, and enhanced sodium retention. Thus, AT1 antagonists are used in the treatment for hypertension and cardiovascular diseases like heart failure, chronic kidney disease, and acute coronary syndrome. Due to the fact that this system is a highly complex hormonal cascade that spans multiple organs and cell types, it becomes evident that it plays an important role in the occurrence of multiple diseases when it malfunctions. Drug repurposing is a convenient way of utilizing existing drugs, such as angiotensin II receptor blockers (ARBs), towards the therapy of different diseases. In this review, we have outlined the importance of drug repurposing (repositioning) for AT1 antagonists and in some cases ACE inhibitors (ACEIs). Examples are given for neurodegenerative diseases, such as Alzheimer’s and Parkinson’s diseases, anxiety, certain types of cancer like glioma, etc. These studies are very promising and, in combination with rational drug design along with the creation of hybrid molecules for the selective inhibition of multiple targets, many new possibilities arise towards the resolution of various pathological conditions. For example, the design of a compound which processes the inhibitory activity of losartan (AT1 inhibitor) and Lanabecestat (a potent BACE1 inhibitor) could open new paths in the treatment of Alzheimer’s disease. Finally, we discuss our own research on novel AT1 antagonists. These molecules range from small non-peptide compounds to sartan derivatives and hybrid molecules. It becomes evident that the possibilities for ARB utilization towards the synthesis or improvement of already existing compounds are many and our research should head towards this goal.

## Figures and Tables

**Table 1:** Representative successful stories of drug repurposing. The disease area, drug name, the drug’s original indication and the new (repositioned) indications, as well as their status, can be seen in the table.

Disease Area	Drug Name/Active Ingredients	Original Indication	New Indication(s)	New Indication(s) Status
*Depression*	Duloxetine hydrochloride	Major Depressive Disorder (MDD) [28,29]	Neuropathic pain [30], generalized anxiety disorder (GAD) [31], osteoarthritis [32], and stress incontinence [33]	Approved
	Fluoxetine hydrochloride	Major Depressive Disorder (MDD) [34]	Premenstrual dysphoric disorder (PMDD) [35]	Approved
*Neurology*	Atomoxetine hydrochloride	Parkinson’s disease (PD) [36]	Attention-deficit hyperactivity disorder (ADHD) [37]	Approved
	Bromocriptine	Parkinson’s disease [38]	Diabetes mellitus [39], prolactinomas, and other pituitary adenomas [40]	Approved
	Chlorpromazine	Schizophrenia, bipolar disorder, and acute psychosis [41]	Breast cancer [42]	Investigational
	Lithium	Depression, bipolar disorder [43]	Cancer [44]	Investigational
	Penfluridol	Chronic schizophrenia, acute psychosis, and Tourette syndrome [45]	Cancer [46]	Investigational
	Ropinirole hydrochloride	Hypertension (HTN) [47]	Parkinson’s disease (PD) [48]	Approved
*Non-neurology*	Aspirin	Analgesic, Antipyretic [49]	Antiplatelet [49], anti-thrombosis [50]	Approved
	Celecoxib	Pain, inflammation, arthritis [51]	Familial adenomatous polyposis [52]	Approved
	Finasteride	Benign prostatic hyperplasia (BPH) [53]	Hair loss [54]	Approved
	Losartan	Hypertension [55]	Dystrophic epidermolysis bullosa (RDEB) [56] and COVID-19 [57]	Investigational
	Minoxidil	Hypertension (HTN) [58]	Hair loss [59]	Approved
	Raltegravir	HIV-1 integrase Inhibitor [60]	Colorectal cancer [61]	Investigational
	Sildenafil	Angina [26]	Erectile dysfunction (ED) and pulmonary arterial hypertension (PAH) [26]	Approved
	Zidovudine	Failed clinical trials for cancer [62]	Human immunodeficiency virus (HIV) [62,63]	Approved
*Cancer*	Auranofin	Rheumatoid arthritis (RA) [64]	Gastrointestinal stromal tumor (GIST) [65]	Investigational
	Crizotinib	Clinical trials for anaplastic large cell lymphoma (ALCL) [66]	Non-small cell lung cancer (NSCLC) [67]	Approved
	Imatinib	Chronic myeloid leukemia (CML) [68]	Gastrointestinal stromal tumors (GIST) [69]	Approved
	Irinotecan hydrochloride	Colorectal cancer [70]	Pancreatic cancer [71]	Approved
	Metformin hydrochloride	Type 2 diabetes (T2DM) [72]	Pancreatic cancer, endometrial cancer, colorectal cancer, and esophageal cancer [73,74,75,76]	Investigational
	Nelfinavir	Human immunodeficiency virus 1 (HIV-1) [77]	Colorectal cancer, lung cancer, cervical cancer, pancreatic cancer, ovarian cancer, metastatic cancer [78]	Investigational
	Raloxifene	Osteoporosis [79]	Breast cancer [80]	Approved
	Rituximab	Various cancers [81]	Rheumatoid arthritis [82]	Approved
	Sunitinib	Renal cell carcinoma (RCC) [83] and Gastrointestinal stromal tumor (GIST) [84]	Pancreatic neuroendocrine tumors (PNETs) [85]	Approved
	Trastuzumab	Human epidermal growth factor receptor 2 (HER2)-positive breast cancer [86]	Metastatic breast cancer, gastric cancer, and early breast cancer [87,88,89]	Approved
	Temsirolimus	Renal cell carcinoma [90]	Lung Adenocarcinoma [91]	Investigational
*Infectious*	Apmphotericin B	Antifungal [92]	Leishmaniasis [93], Mucormycosis [94]	Approved
	Dapoxetine	Premature ejaculation [95]	Zika virus infection [96]	Investigational
	Everolimus	Immunosuppressant [97]	Pancreatic neuroendocrine tumors (PNETs) [98], renal cell carcinoma (RCC) [99], and subependymal giant cell astrocytoma (SEGA) [100]	Approved
	Favipiravir	Influenza [101]	SARS-CoV-2 [102]	Investigational
	Ivermectin	Antiretroviral [103]	SARS-CoV-2 [104]	Investigational
	Ketoconazole	Fungal infections [105]	Cushing’s syndrome [106]	Approved
	Remdesivir	Antiviral [107]	SARS-CoV-2 [107]	Approved
	Sirolimus	Organ rejection in patients receiving renal transplants [108]	Malaria [109]	Investigational
	Thalidomide	Morning sickness (withdrawn) [67]	Erythema nodosum leprosum (Leprosy) [110] and Multiple Myeloma [111]	Approved
*Rare and orphan*	Alefacept	Chronic plaque psoriasis [112]	Memory T cell-mediated autoimmune diseases, organ transplantation and type I diabetes (T1D) [113]	Investigational
	Baclofen	Muscle relaxant [114]	Alcohol use disorder [115]	Approved
	Clobetasol	Psoriasis [116]	post-cataract surgery pain and inflammation [117]	Approved
	Dimethyl fumarate	Multiple sclerosis [118]	Psoriasis [119], anti-inflammatory [120]	Approved
	Erythropoietin	Anemia [121]	Traumatic brain injury [122]	Investigational
	Ethinyl estradiol	Turner syndrome [123]	Contraceptive applications [124], acne, hirsutism [125]	Approved
	Fingolimod	Multiple sclerosis (MS) [126]	Alzheimer’s disease [127], cancer [128], diabetic retinopathy [129]	Investigational
	Liraglutide	Diabetes [130]	Alzheimer’s disease (AD) [131]	Investigational
	N-acetyl cysteine	Mucolytic agent [132]	Obsessive–compulsive disorder [133]	Investigational
	Pregabalin	Neuropathic pain [134]	Generalized anxiety disorder [135]	Approved
	Simvastatin	Cardiovascular diseases [136]	Anti-tumor agents [137], neurodegenerative disorders [138], preterm labor [139] and Klebsiella pneumoniae infections [140]	Investigational
	Topiramate	Epilepsy [141]	Obesity [142]	Approved

**Table 2:** A summary of the diseases, the mechanistic rationale, and the involvement of the RAAS in their development, as well as the preclinical and clinical data collected for these diseases/indications from Section 3.

Disease	RAAS Involvement	Preclinical/Clinical Support	Example ARB(s)
*Hypertension*	Antagonism with Ang II for the active site of the AT1 receptor; lower blood pressure	ARBs; approved drugs for hypertension	All sartans (losartan and its active metabolite EXP3174, olmesartan, eprosartan, irbesartan, telmisartan, candesartan)
*Heart Failure*	ARBs downstream action; blockade of Ang II from AT1 receptors; Ang II binding to AT2 receptors	ARBs approved for patients with HFrEF (Class I) and HFmrEF (Class IIa) who are intolerant to ACEIs and ARNI	Candesartan [253], valsartan [254]
*Chronic Kidney Disease*	Ang II through AT1R activates signaling cascades—MAPK/ERK, JNK, STAT, NF-κB, and AP-1; drives fibrosis, inflammation, cell proliferation; Ang II causes cytoskeletal disruption, ROS production, apoptosis, podocytopathy, and glomerulosclerosis	ARBs for patients with CKD and severely increased albuminuria (1B), moderately increased albuminuria (2C) and moderate-to-severe albuminuria and diabetes (1B)	Losartan [255], irbesartan [256], and telmisartan [257]
*Acute Coronary Syndrome*	RAS activation worsens outcomes post–MI; Ang II activates NADPH oxidase, causing oxidative injury and atherosclerosis; Ang II, AT1R, and ACE co-localize in plaques, promoting IL-6 release and instability of ARBs post-MI upregulation	ARBs for patients with intolerance of ACE inhibitors, after ACS with HF symptoms, LVEF < 40%, hypertension, and/or CKD	Valsartan [258], losartan [259], candesartan [260]
*Alzheimer’s Disease*	Ang II via AT1R increases Aβ through the upregulation of APP mRNA, β-secretase activity, and presenilin expression; promotes tau phosphorylation and ROS generation, neuroinflammation, oxidative stress, neurotoxicity	Candesartan: safe and reduces brain amyloid biomarkers, improves subcortical brain connectivity, and supports cognitive function in non-hypertensive individuals with prodromal Alzheimer’s disease	Telmisartan, candesartan, losartan, and irbesartan
*Parkinson’s Disease*	Regulates dopaminergic neurotransmission, blood flow, inflammatory responses; intricate interaction between Ang and DA; balance of D1, D2, AT1, AT2 receptors.	Animal models of PD; neuroprotective effects of AT1R antagonists, effects driven by decrease in ROS; perindopril enhanced the effects of levodopa without causing dyskinesias	Losartan, candesartan, and telmisartan
*Anxiety*	Overactivation of RAS through AT1R; HPA axis hyperactivity; anxiety-like behaviors; Ang II via AT1R stimulates CRH production, AVP release, adrenal catecholamine output, amplifying stress responses	Clinical study: ARB-treated patients had lower anxiety STAI scores than those on ACEIs or drug-free at baseline and during the follow-up	Losartan, telmisartan [261]
*Cancer Glioma*	Glioblastoma cells express renin, angiotensinogen, renin receptor, ACE, AT1R, AT2R, renin inhibition induces apoptosis, Ang (1–7) inhibited the JNK pathway; preserved endothelial junction integrity, reduced vascular leak, and limited tumor-induced edema.	Phase I clinical trial; patients with glioblastoma treated with combination of RAS modulators, treatment well tolerated; preserves quality of life/performance; may lengthen survival time	Losartan [262], telmisartan
*Pathogenic Inflammation*	Candesartan suppressed the NLRP3 inflammasome and pyroptosis in mac-rophages, reduced the expression of NLRP3 and proIL-1β by inhibiting NF-κB activation and decreasing phosphorylation of ERK1/2 and JNK1/2, lessened mitochondrial damage	A meta-analysis of randomized controlled trials found that ARBs significantly reduced levels of inflammatory markers such as CRP, IL-6, and TNF-α	Candesartan
*Candidosis*	Medications targeting the RAAS inhibit secreted aspartic proteases produced by *Candida albicans*	Preclinical studies; ARBs disrupt the metabolism and formation of *Candida* biofilms; decreased fungal viability at all concentrations	Losartan [223], candesartan [263]
*Fibrosis*	Ang II through AT1R, TGF-β/Smad activation; ROS, inflammation; Ang (1–7) through MAS receptor inhibits fibrosis, reduces inflammation, restores tissue integrity; liver fibrosis; AT2R upregulated; antifibrotic effects by inhibition of the IRE1α-XBP1 pathway	Ongoing clinical trials; ARBs in reducing fibrosis in sickle cell disease (NCT05012631), aortic stenosis (NCT04913870), AKI (NCT05272878), patients with hepatitis C and hypertension, ARB treatment, reduced fibrosis relative to untreated patients.	Losartan [264], telmisartan [265], candesartan [266], valsartan [267]
*Tissue Fibrosis in Systemic Sclerosis*	Ang II, fibrosis through ECM stimulation	Clinical study: compares the efficacy and tolerability of losartan, with nifedipine for the treatment of primary and secondary RP; tolerability of short-term treatment of RP with losartan	Losartan
*Diabetic Peripheral Neuropathy*	Spinal Ang II/AT1R signaling drives neuropathic pain via p38 MAPK; losartan blockade, Ang II–driven ROS via NADPH oxidase; broader neurotoxicity of Ang II/AT1R pathway	Clinical study: Bonferroni’s test indicated significantly later DPN development in the ARB and ACEI groups, beneficial to prevent DPN accompanying T2DM	Losartan [268], telmisartan [269]
*Inflammatory Bowel Diseases*	Ang II; activation of RAS; activation of JAK2/STAT1/3, elevated TH1/TH17 T-cell responses, IEC apoptosis, Ang (1–7)/MASR reduces signaling (p38/ERK/Akt)	Retrospective studies of IBD patients treated with ACEIs or ARBs; encouraging results, including milder disease progression, fewer hospitalizations, reduced corticosteroid use	Losartan [270], telmisartan [271], candesartan [272], valsartan [273]
*Marfan Syndrome*	AT_1_R blockade in MFS mice; prevents aneurysm, reverses pathology via TGF-β/Smad suppression, AT2R; pivotal role for full therapeutic effect; required for ERK inhibition	Individuals with Marfan syndrome who have not undergone aortic surgery; ARBs reduced the rate of aortic root Z score enlargement by roughly half, even in those also taking beta-blockers.	Losartan [274], irbesartan [275], telmisartan [276]
*SARS-CoV-2*	ARBs increase levels of ACE2 more than other hypertension medications, ACE2 entry point for SARS-CoV-2 in the nasopharynx, lungs, and heart cells; ACE2 transforms harmful Ang II into the beneficial peptides Ang (1–7) and alamandine, helps maintain balance while simultaneously preventing SARS-CoV-2 from entering through ACE2.	Phase III clinical trial “CLARITY”; no evidence of benefit, based on disease severity score, for treatment with ARBs, seamless phase I and II study; intravenous infusion Angiotensin (1–7) in COVID-19 patients admitted to the ICU with severe pneumonia; in Phase II; no significant difference in OFD between groups (premature termination, further studies need to be performed)	Telmisartan [277], candesartan [278]
*Rheumatoid Arthritis*	Ang II increases IL-1, IL-6, TNF-α; role in the development of RA	Clinical research indicates that RAS inhibitors—especially ARBs, as well as ACE inhibitors and renin inhibitors—play a role in RA by primarily targeting inflammation and oxidative stress.	Losartan [279], olmesartan, candesartan, telmisartan [280]
*Osteoarthritis*	Joint tissues; AT1R stimulation by Ang II or inflammatory cytokines (IL-1β) triggers the release of pro-inflammatory substances and MMPs, speeding up cartilage degradation and wors-ening joint injury.	No clinical trials performed; one trial in patients with OA and hypertension; valsartan with strong osteoarthritis adverse reaction signals compared to irbesartan, cloxartan	Losartan [281]
*Opioid Addiction*	ARBs with higher affinities for AT1R demonstrate higher affinities for µORs and ժORs than opiate ligands, such as fentanyl and naltrexone	No ongoing clinical trials	Telmisartan [282], candesartan [283], valsartan

## Abbreviations

The following abbreviations are used in this manuscript:

RAAS	Renin–Angiotensin–Aldosterone system (RAAS)
Ang I	Angiotensin I
Ang II	Angiotensin II
ACE	Angiotensin-converting enzyme
AT1R	Angiotensin II Type 1 receptor
AT2R	Angiotensin II Type 2 receptor
MTDLs	Multi-target Directed Ligands
HT	Hypertension
HF	Heart Failure
CKD	Chronic Kidney disease
ACS	Acute Coronary Syndrome
AD	Alzheimer’s disease
PD	Parkinson’s disease
RA	Rheumatoid Arthritis
OA	Osteoarthritis
DN	Diabetic Nephropathy
DPN	Diabetic Peripheral Neuropathy
SARS-CoV-2	Severe Acute Respiratory Syndrome Coronavirus 2
CS	Candidosis
FS	Fibrosis
TFSSc	Tissue Fibrosis in Systemic Sclerosis
RP	Raynaud’s phenomenon
GC	Glioma Cancer
EAE	Auto-immune encephalomyelitis
MS	Marfan Syndrome
MBP	Myelin Basic Protein
IBDs	Inflammatory Bowel diseases
TH17	T helper 17
Akt	Protein kinase B
ICU	Intensive Care Unit
AT	Anxiety
PI	Pathogenic Inflammation
YU	Yet Unknown diseases
UK	United Kingdom
PDE5	Phosphodiesterase-5
cGMP	Cyclic Guanosine Monophosphate
NO	Nitric Oxide
CAD	Coronary Artery disease
CRS	Charge Relay System
ARBs	Angiotensin II receptor blockers
CCBs	Calcium channel blockers
ARNIs	Angiotensin receptor neprilysin inhibitors
ESC	European Society of Cardiology
HFrEF	Heart Failure with Reduced Ejection Fraction
HFmrEF	Heart Failure with Mid-Range Ejection Fraction
KDIGO	Kidney Disease: Improving Kidney Outcomes
APP	Amyloid precursor protein
Aβ	Amyloid-beta
BACE1	Beta-site amyloid precursor protein-cleaving enzyme 1 inhibitors
CSF	Cerebrospinal fluid
AT4	Angiotensin IV receptor
ACEIs	Angiotensin-converting enzyme inhibitors
Ang	Angiotensin
DA	Dopamine
ROS	Reactive Oxygen Species
STAI	State-Trait Anxiety Inventory
BBB	Blood–Brain Barrier
FDA	Food and Drug Administration
U87	Uppsala 87 Malignant Glioma
PCR	Polymerase Chain Reaction
TNF-α	Tumor necrosis factor alpha
IL-6	Interleukin-6
NLRP3	NOD-like receptor family pyrin domain containing 3
proIL-1β	pro-interleukin-1β
NF-κB	Nuclear factor kappa light chain enhancer of activated B cells
ERK1/2	Extracellular signal-regulated kinase 1 and 2
JNK1/2	c-Jun N-terminal kinase 1 and 2. to PKR, NEK7, and ASC.
AP-1	Activator Protein-1
LVEF	Left Ventricular Ejection Fraction
PKR	Protein kinase R
NEK7	NIMA-related kinase 7
ASC	Apoptosis-associated speck-like protein containing a CARD
T2DM	Type 2 diabetes mellitus
FBN1	Fibrillin-1
Ang (1–7)	Angiotensin (1–7)
IL-1	Interleukin-1
MMPs	Matrix metalloproteinases
ECM	Extracellular matrix
TGF-β	Transforming Growth Factor-β
CVD	Cardiovascular disease
NADPH	Nicotinamide Adenine Dinucleotide Phosphate
MAS	MAS proto-oncogene
IRI	Ischemia–Reperfusion Injury
CRH	Corticotropin-releasing hormone
AVP	Arginine Vasopressin
STAT3	Signal transducer and activator of transcription 3
MAPK	Mitogen-activated protein kinase
CD	Crohn’s disease
UC	Ulcerative colitis
VEGF	Vascular endothelial growth factor
Caspase-3	Cysteine-aspartic acid protease 3
IC50	Half-maximal inhibitory concentration
GBM	Glioblastoma multiforme
PBMC	Peripheral Blood Mononuclear Cells
MHC II	Major Histocompatibility Complex Class II molecule
Smad	Suppressor of Mothers against Decapentaplegic
IRE1α-XBP1	Inositol-Requiring Enzyme 1 alpha-X-Box Binding Protein 1
AKI	Acute Kidney Injury
DHA	Docosahexaenoic acid
P2Y12	Purinergic Receptor P2Y, G-protein coupled 12
PAR-1	Protease-Activated Receptor-1
PAF	Platelet-Activating Factor
COX-1	Cyclooxygenase-1
MD	Molecular Dynamics
GPCRs	G protein-coupled receptors
α1AR	α1-adrenergic receptor
α2AR	A2-adrenergic receptor
µ-(µOR)	μ-opioid receptor
ժOR	δ-opioid receptor
